# Insidious Cases of Enlarged Vestibular Aqueduct (EVA) Syndrome Resembling Otosclerosis: Clinical Features for Differential Diagnosis and the Role of High-Resolution Computed Tomography in the Pre-Operative Setting

**DOI:** 10.3390/audiolres14040050

**Published:** 2024-06-27

**Authors:** Giovanni Motta, Salvatore Allosso, Ludovica Castagna, Ghita Trifuoggi, Tonia Di Meglio, Domenico Testa, Massimo Mesolella, Gaetano Motta

**Affiliations:** 1 ENT Unit-Department of Mental, Physical Health and Preventive Medicine, University of Campania “Luigi Vanvitelli”, 80121 Naples, Italy; ludovica.castagna@studenti.unicampania.it (L.C.); ghita.trifuoggi@studenti.unicampania.it (G.T.); tonia.dimeglio@studenti.unicampania.it (T.D.M.); domenico.testa@unicampania.it (D.T.); gae.motta@libero.it (G.M.); 2 Otorhinolaryngology-Head and Neck Surgery Unit, Department of Neuroscience, Reproductive and Odontostomatological Sciences, University of Naples Federico II, 80138 Naples, Italy; salvatore.allosso@unina.it (S.A.); massimo.mesolella@unina.it (M.M.)

**Keywords:** enlarged vestibular aqueduct (EVA), otosclerosis, air–bone gap (ABG), conductive hearing loss (CHL), mixed hearing loss (MHL), third mobile window (TMW), gusher syndrome, dead ear, stapes surgery

## Abstract

Background: Enlarged vestibular aqueduct (EVA) syndrome can mimic otosclerosis in adults, presenting with an air–bone gap (ABG) and even absent stapedial reflexes. The ABG in inner-ear disorders is currently the object of several authors’ studies and seems to be related to a third mobile window (TMW) phenomenon. This can lead to misdiagnosis and inappropriate treatment. Given that it would be inappropriate and harmful to perform CT scans in all patients with a clinical diagnosis of otosclerosis, this study aims to highlight some clinical features useful for the differential diagnosis between otosclerosis and these rare cases of EVA presenting with an ABG, thus enabling the identification of suspected cases to be tested with CT scans. Methods: Between April and May 2024, a narrative review was conducted focusing on the differential diagnosis between some rare cases of EVA and otosclerosis. Clinical, audiological, and radiologic features of both conditions were investigated. Results: This review demonstrates the diagnostic challenge in differentiating atypical cases of EVA from otosclerosis in a subset of patients. Clinical and audiological features are important for differential diagnosis, but may not always be sufficient. Therefore, high-resolution computed tomography (HRCT) of the temporal bone plays a pivotal role in definitive diagnosis. Conclusions: In some specific cases, pre-operative imaging assessment using HRCT emerges as an essential tool for differentiating these two conditions and avoiding unnecessary stapes surgery.

## 1. Introduction

Enlarged vestibular aqueduct (EVA) syndrome can mimic otosclerosis in adults by presenting with conductive or mixed progressive hearing loss and even an absent stapedial reflex [1,2], which are the typical features of otosclerosis. With it being an inner-ear disorder, patients with EVA typically display sensorineural hearing loss. However, cases of EVA with conductive and mixed hearing loss have also been described. The conductive component in inner-ear disorders is currently the object of several studies among many authors and it seems to be related to a third window phenomenon. This can lead to misdiagnosis and inappropriate treatment, such as stapes surgery, with the risk of inducing a “gusher” or profound neurosensorial hearing loss. This happens because EVA, unlike otosclerosis, is associated with a “gusher”, where perilymph followed by cerebrospinal fluid exits the stapedotomy [1,2,3,4,5,6,7,8].

Therefore, it is crucial for clinicians to differentiate between these rare cases of EVA and otosclerosis, particularly in patients born before widespread implementation of newborn hearing screening programs. Misdiagnosis can lead to inappropriate treatment and potentially severe consequences for the patient’s hearing [1,2,3,4,5,6,7,8,9].

Given that it would be inappropriate and harmful to perform CT scans for all patients with a clinical diagnosis of otosclerosis, this study aims to highlight some clinical features useful for the differential diagnosis between otosclerosis and these rare cases of EVA presenting with conductive or mixed hearing loss, thus allowing the identification of suspected cases for testing with CT scans of the petrous bones.

## 2. Materials and Methods

A narrative review was conducted between April and May 2024 to investigate the challenges in differentiating atypical cases of EVA from otosclerosis. 

This review focused on the differential diagnosis between otosclerosis and EVA in rare and complex cases by excluding other inner-ear disorders that can occasionally present with an air–bone gap and middle- and external-ear pathologies. Therefore, only patients presenting with atypical EVA were included. These atypical EVA cases were specifically selected based on their clinical and audiological features that demonstrably overlapped with those characteristics of otosclerosis. The review also explored the role of pre-operative high-resolution computed tomography (HRCT) in insidious cases.

The keywords used were enlarged vestibular aqueduct (EVA), otosclerosis, air–bone gap (ABG), conductive hearing loss (CHL), mixed hearing loss (MHL), third mobile window (TMW), gusher syndrome, dead ear, and stapes surgery. 

## 3. Results

The review highlighted that the differential diagnosis between atypical EVA and otosclerosis can be challenging in some rare and complex cases (Table 1). Careful clinical and audiological evaluation is important for differentiating rare and atypical cases of EVA from otosclerosis. However, these rare cases of EVA can sometimes present with overlapping clinical and audiological features, making accurate differentiation challenging (Table 1). Consequently, pre-operative imaging assessment using high-resolution computed tomography (HRCT) of the temporal bones emerges as an essential and decisive tool for identifying these two conditions and avoiding unnecessary stapes surgery or middle-ear explorations (Table 2). Table 3 summarizes the findings reported in the literature by the authors included in this review. By describing cases of EVA that closely resembled otosclerosis in their clinical and audiological presentations, the detrimental consequences of misdiagnosing EVA as otosclerosis are highlighted.

## 4. Discussion

### 4.1. Definition and Pathophysiology of TMW Syndromes

The presence of inner-ear disorders that manifest with hearing loss that appears to be conductive, and therefore require differential diagnosis with middle-ear pathologies, has been investigated and is currently the object of several authors’ research [1,2,4,9]. However, since these conditions are not dependent on conduction phenomena in the middle ear but rather on phenomena related to a TMW, it is more appropriate to speak of ABGs when referring to one of these conditions and not of CHL, since the hearing loss is not due to mechanical and/or inflammatory conduction problems in the external and middle ear. This terminology more accurately conveys the audiometric finding of a difference between air and bone conduction thresholds, regardless of the source of the gap. Many authors have stressed the importance of this distinction since the ABG in TMW syndromes is usually due to abnormalities of the inner ear. Indeed, upon clinical examination, these patients present with intact tympanic membranes and completely healthy and aerated middle-ear cavities [9,10,16]. The presence of an additional window in the inner ear can lead to substantial ABGs at low frequencies, posing diagnostic and treatment challenges in various conditions. Several authors have proposed a physiopathological explanation for the ABG in TMW syndromes. Normally, sound waves enter the inner ear through the oval window, and the round window acts as an outlet for the vibrations within the cochlea, ensuring efficient sound transmission. However, when a TMW exists, it introduces an additional pathway for sound energy to escape from the inner ear (the scala vestibuli) before it can be fully sensed by the cochlea. This escape of sound energy leads to a reduction in the effectiveness of air-conducted sound transmission, thereby increasing the air conduction threshold, especially at low frequencies. This phenomenon is observed as a loss in hearing sensitivity for air-conducted sounds, or an increase in the air conduction threshold. On the other hand, bone-conducted sound waves, which bypass the outer and middle ear to reach the cochlea directly, are less affected by this additional window. In fact, the presence of a third window can decrease the impedance (resistance to sound energy flow) on one side of the cochlear partition, improving the cochlea’s response to bone-conducted sounds. This differential effect on air and bone conduction leads to an apparent air–bone gap on audiometric testing, where the measured bone conduction thresholds are better (lower) than the air conduction thresholds, particularly at low frequencies [1,2,4,5,9,10,16]. Among the most well-known and prevalent TMW syndromes are superior semicircular canal dehiscence (Minor’s syndrome) and EVA syndrome. In these conditions, the development of the pathological third window can be attributed to the thinning of the otic capsule’s bony components or to an enlargement of the vestibular aqueduct [9,16].

### 4.2. Overlapping Clinical–Audiological Features between Atypical Cases of EVA and Otosclerosis

In more detail, many authors have highlighted that EVA and middle-ear pathologies can present with overlapping clinical features, posing a significant challenge for differential diagnosis. This diagnostic complexity is particularly evident in atypical and insidious cases of EVA, which can mimic classic otosclerosis, leading to misdiagnosis and potentially severe consequences for patients. In a paper published in 2013 by Wiekzorek et al., the authors presented three atypical cases of EVA syndrome in adults, which closely mimicked the clinical and audiological features of otosclerosis. The resemblance to otosclerosis was primarily due to the presence of progressive hearing loss and absent stapedial reflexes in all three patients. Additionally, the mixed hearing loss observed in these patients further complicated the clinical picture, as otosclerosis can also present with mixed hearing loss in advanced stages [1,17]. In a case report published by Távora-Vieira and Miller in 2012, the authors reported that the misdiagnosis and mismanagement of a case of EVA syndrome led to profound hearing loss. The patient was initially misdiagnosed with otosclerosis and underwent a stapedectomy, which resulted in a loss of hearing in their right ear. In line with the previous cases, stapedial reflexes were also absent in this patient [2]. Another similar case was published by Shirazi et al. in 1994: this case involved a 21-year-old female diagnosed with EVA and concurrent stapes fixation. During a stapedectomy procedure aimed at addressing her hearing loss, a perilymphatic gusher occurred, highlighting the risks associated with such surgeries in the presence of an EVA [3]. Another interesting case useful for differential diagnosis was recently published by Yang et al. in 2023. The authors described the case of a 39-year-old female patient presenting with a 3-year history of right hearing loss, with no history of head trauma or otitis media. Ear endoscopy showed bilateral normal external auditory canals and intact tympanic membranes. The Rinne test was negative on the right side and positive on the left, with Weber’s test lateralizing to the right ear. Gelle’s test was positive in the right ear. Pure-tone audiometry revealed CHL with an ABG of 30 to 40 dB in the right ear, while the left ear had a normal audiometric threshold. Acoustic immittance testing showed bilateral As-type tympanograms, with the presence of ipsilateral and contralateral stapedial reflexes in the right ear, indicating normal middle-ear conduction. The patient’s clinical presentation initially suggested otosclerosis. However, several findings contradicted this diagnosis and pointed towards an alternative cause. Firstly, the presence of acoustic reflexes and a positive Gelle’s test indicated that the stapes was not fixed, which is inconsistent with otosclerosis. Additionally, the pure-tone threshold curve lacked a Carhart notch. High-resolution computed tomography (HRCT) of the temporal bone revealed an EVA on the right side, leading to the correct diagnosis and avoiding unnecessary surgery [4]. Another recent paper by Lorente-Piera et al. reported one more case of EVA resembling otosclerosis. Their case involved a 46-year-old male presenting with a six-week history of instability, auditory fluctuations, and ear fullness in the left ear. Otoscopic and neurotological examinations were normal. Audiometry revealed mild hearing loss with a conductive component in the left ear. The vestibular head impulse test (vHIT) showed no abnormalities, but ocular-vestibular-evoked myogenic potentials (VEMPs) indicated utricular hypofunction in the left ear. The patient did not meet the criteria for Ménière’s disease. With the suspicion of otosclerosis and possible retrofenestral involvement, a temporal bone CT scan was requested. The scan revealed a dehiscence at the level of the jugular diverticulum with the vestibular aqueduct of the left endolymphatic sac, along with a dehiscence also in that ear at the level of the superior semicircular canal [8]. Another five cases were described by Merchant et al. in 2007. In their study, patients with EVA mimicking otosclerosis were highlighted through a detailed examination of patients presenting with ABGs. These cases were attributed to EVA based on specific clinical, audiological, and imaging features that argued against otosclerosis. Firstly, these patients did not present with the typical progressive hearing loss characteristic of otosclerosis. Instead, some had had stable hearing loss since birth or experienced sudden idiopathic sensorineural hearing loss. Otoscopic examinations revealed normal tympanic membranes and ear canals, and exploratory tympanotomy in one case showed an intact and mobile ossicular chain with a patent round-window niche, arguing against middle-ear disease. Audiometry in these cases showed a large low-frequency ABG. The presence of acoustic reflexes, VEMP responses, and distortion product otoacoustic emissions (DPOAEs) in some cases further argued against middle-ear disease. CT scans were crucial in the differential diagnosis, showing large vestibular aqueducts in the affected ears without stapedial footplate alterations, which are typically seen in otosclerosis [10,17,18,19]. As emerges from the cited literature, atypical cases of EVA and otosclerosis can pose a significative diagnostic challenge due to the possibility of overlapping clinical and audiological features (ABGs at low frequencies, same state of stapedial reflexes, and normal middle-ear function), making differential diagnosis challenging [1,2,4,5,10,16]. 

### 4.3. The Role of High-Resolution Computed Tomography (HRCT)

These overlapping features can make it difficult to distinguish atypical EVA from otosclerosis based on clinical and audiological evaluation alone. Consequently, high-resolution computed tomography (HRCT) of the temporal bones emerges as a crucial tool for differential diagnosis [6,7,9,10,16]. In this context, as McElveen and Kutz pointed out in 2018, the role of pre-operative CT in the evaluation of suspected otosclerosis remains controversial. While some surgeons routinely obtain pre-operative CT scans for all patients with suspected otosclerosis, citing their ability to identify inner-ear abnormalities and predict surgical outcomes and risks, there are concerns regarding radiation exposure and costs associated with these scans. Specifically, the risk of CT-related malignancy, though extremely low, does exist, and the costs of CT scans in countries like the USA can be significant. Despite these concerns, CT scans can play a crucial role in atypical cases where there is a suspicion of conditions that could mimic otosclerosis, such as EVA [6]. In support of this, in a 2018 paper by Wolfovitz and Luntz, the authors highlighted the crucial role of pre-operative imaging in preventing complications during stapes surgery. Specifically, HRCT should be performed prior to stapes surgery, especially if the patient has a history of failed contralateral stapedectomy or stapedotomy [7]. This imaging modality could prevent serious complications by revealing inner-ear disorders, such as EVA, that could compromise the outcomes of such surgery [2,3,7]. Regardless, it is undeniable that complications such as gusher syndrome and dead ear represent a significant issue with serious implications for patients’ lives [2,3,11,20]. Therefore, these complications should be actively sought out and identified to prevent stapes surgery before they occur in the contralateral ear [2,3]. Based on the cases presented in the literature, it would be prudent to incorporate pre-operative HRCT of the temporal bones if there is the occurrence of one or more of the following situations: patients with a history of hearing loss since childhood, especially in the absence of a family history of otosclerosis [1,3,11]; a low-frequency ABG with supranormal thresholds for bone conduction [4,8,17]; the presence of stapedial reflexes [4,10]; suspicious EKG with prolonged QT intervals, as these may indicate genetic cardiac conditions such as a possible Jervell and Lange-Nielsen syndrome, especially if the patient reveals a family history of sudden cardiac death or a personal history of syncopal episodes [1,12,13]. In such cases, which can be very variable in their clinical presentations, genetic evaluation should precede the radiological examination of the temporal bones. If this syndrome is confirmed, cochlear implantation should be considered for hearing rehabilitation [12,13]. Ultimately, patients with thyroid anomalies and hearing loss should undergo genetic testing and HRCT of their temporal bones, given Pendred syndrome (PDS)’s variable clinical picture. PDS is a genetic disorder characterized by hearing loss with variable presentations (ranging from mild to profound, progressive, fluctuating, or sudden) and thyroid anomalies [14,15]. As demonstrated by Forlì et al., hearing loss in PDS is not exclusively sensorineural but can also be mixed, with an ABG. This finding was observed in 21 out of 66 patients in their study. This variability in the auditory presentation is partly attributed to associated inner-ear malformations, such as EVA with or without Mondini dysplasia [14,15]. Therefore, in patients that may be syndromic, genetic evaluation plays a crucial role together with imaging in order to establish the most appropriate management of each case. Given the complex nature of these conditions, otologists who suspect the presence of one of these conditions are strongly encouraged to seek consultation with a geneticist to ensure accurate patient assessment and management [12,14,15]. 

Further research focused on the genetic, clinical, and audiological characteristics of these rare EVA cases is warranted to optimize diagnostic and therapeutic approaches for the affected patients. Until the mechanisms underlying TMW syndromes are fully elucidated, pre-operative imaging should be recommended for the evaluation of complex and insidious cases. Moreover, the recommendation of pre-operative imaging in equivocal cases is even more compelling for young surgeons. This is because the interpretation of radiological findings is now essential for the training of young oto-surgeons, and also because a younger surgeon would have more difficulty managing a complication such as a gusher compared to an older surgeon with more experience [21,22,23].

## 5. Conclusions

This narrative review provides an overview of the differential diagnosis between rare and insidious cases of EVA and otosclerosis. Implementing the aforementioned recommendations, including the use of pre-operative CT in suspected cases, together with genetic testing in potentially syndromic patients, could help to enhance diagnostic accuracy and prevent serious complications such as gusher syndrome and dead ear, which can occur during stapes surgery if atypical cases of EVA are not correctly identified.

## Figures and Tables

**Table 1:** Clinical, audiological, and imaging features of atypical cases of EVA and otosclerosis.

Feature	Atypical EVA	Otosclerosis
Hearing Loss	Not always progressive. May be stable from birth or sudden sensorineural hearing loss.	Progressive conductive hearing loss. Usually not present from childhood.
Stapedial Reflexes	Usually present, but may be absent.	Usually absent or on/off.
Pure-Tone Audiometry	Large, low-frequency air–bone gap (ABG). May lack the Carhart notch.	Conductive or mixed hearing loss with a possible Carhart notch.
Tinnitus	May be present.	May be present.
Vertigo	May be present.	Uncommon.
Otoscopic Examination	Normal tympanic membranes and ear canals.	Normal tympanic membranes and ear canals. Schwartze sign may be present.
Inner-Ear Imaging	Large vestibular aqueduct on high-resolution CT (HRCT) scan.	Normal inner-ear structures or osteosclerotic changes on CT scan.
Gelle Test Findings	Usually abnormal, with absent or decreased bone conduction response.	Usually normal bone conduction response.
Management	Conservative management (hearing aids, etc.) or cochlear implantation.	Stapes surgery may be considered.
Risk of Misdiagnosis	High (can mimic otosclerosis).	Low.

**Table 2:** One or more of these situations may require pre-operative HRCT assessment.

HRCT
1. History of hearing loss dating back to childhood, especially in cases of absence of familiarity for otosclerosis [1,3,10,11]
2. Low-frequency air–bone gap with supranormal thresholds for bone conduction [1,4,8,10]
3. Presence of stapedial reflexes [4,10], although absent reflexes have been reported [1,2]
4. Previous gusher and/or dead ear following stapes surgery or unsuccessful contralateral stapes surgery [2,3,7,11]
5. Suspicious EKG with prolonged QT interval (possible Jervell and Lange-Nielsen syndrome) [1,12,13]
6. Mixed hearing loss and thyroid alterations (possible Pendred syndrome) [14,15]

**Table 3:** Results reported in the literature regarding cases of EVA resembling otosclerosis.

Author	Symptoms	Audiological Findings	Study Results
Wiekzorek et al., 2013 [1]	Progressive bilateral hearing loss since childhood (patient 1); progressive bilateral hearing loss (patient 2); progressive bilateral hearing loss and pulsatile tinnitus on the right side (patient 3)	Mixed hearing loss, absent stapedial reflexes, and normal tympanometry in all three patients.	Atypical EVA can mimic the clinical and audiological features of otosclerosis, potentially leading to misdiagnosis.
Távora-Vieira and Miller, 2012 [2]	Profound hearing loss in the right ear following a stapedectomy, moderate to severe hearing loss in the left ear, tinnitus in the right ear, imbalance	Moderate to severe sensorineural hearing loss in the left ear and profound sensorineural hearing loss in the right ear, absent ipsilateral and contralateral acoustic reflexes bilaterally, poor word discrimination in quiet conditions using AB word list, significant canal paresis in her right ear as revealed by caloric testing.	Misdiagnosis of EVA as otosclerosis can lead to unnecessary surgery and hearing loss.
Shirazi et al., 1994 [3]	Right-side progressive hearing loss present since early childhood	Moderate to severe mixed hearing loss in the right ear, with Weber’s test lateralizing to the right ear.	Stapedectomy for EVA can lead to perilymph gusher.
Yang et al., 2023 [4]	Three-year history of right hearing loss	Hearing loss with air–bone gap (ABG) of 30 to 40 dB in right ear; tuning-fork tests indicated a negative Rinne’s test on the right side and positive on the left, with Weber’s test lateralizing to the right. Gelle’s test was positive in the right ear. Tympanometry showed an As tympanogram bilaterally. Presence of ipsilateral and contralateral stapedial reflexes in the right ear.	High-resolution CT scans are crucial for differentiating EVA from otosclerosis and avoiding unnecessary surgery.
Lorente-Piera et al., 2023 [8]	Six-week history of instability, auditory fluctuations, and ear fullness in left ear	Hearing loss with a mild air–bone gap (ABG) in the left ear. Absent stapedial reflex on the left side. The vestibular head impulse test (VHIT) showed no abnormalities, but ocular vestibular-evoked myogenic potentials (VEMPs) indicated utricular hypofunction in the left ear.	Atypical EVA can present with symptoms similar to otosclerosis.
Merchant et al., 2007 [10]	Bilateral stable hearing loss since birth (patient 1); long-standing left-sided hearing loss, tinnitus, and aural fullness (patient 2); congenital hearing loss on the left and sudden idiopathic hearing loss on the right at the age of 40 (patient 4); long-standing left-sided hearing loss, tinnitus, and aural fullness for about a year (patient 5)	Large low-frequency air–bone gap (ABG) in all patients. Patient 1 showed normal word recognition scores and tympanometric findings; acoustic reflexes were present on the right and absent on the left; VEMPs were present and normal on both sides; high normal umbo velocity. Patient 2 showed normal recognition scores and normal tympanometry and acoustic reflexes present in both ears; DPOAEs present on the right but absent on the left; normal umbo velocity on both sides. Patient 4 showed word recognition scores of 62% on the right and 4% on the left; umbo velocity was normal on the right, but higher than the mean normal on the left. Patient 5 showed normal word recognition scores; normal tympanometry; acoustic reflexes present in both ears; DPOAEs present on the right but absent on the left; normal umbo velocity on both sides.	CT scans are essential for differentiating EVA from otosclerosis as they can reveal large vestibular aqueducts.
